# Postpartale Depression – wer kümmert sich? Versorgungszugänge über Hebammen, Gynäkologie, Pädiatrie und Allgemeinmedizin

**DOI:** 10.1007/s00103-022-03545-8

**Published:** 2022-05-12

**Authors:** Silke Pawils, Eileen Kochen, Nora Weinbrenner, Viola Loew, Kornelia Döring, Daria Daehn, Claudia Martens, Philip Kaczmarek, Babette Renneberg

**Affiliations:** 1 Zentrum für Medizinische Psychologie, Institut und Poliklinik für Medizinische Psychologie, Universitätsklinik Hamburg-Eppendorf, Martinistraße 52, 20246 Hamburg, Deutschland; 2 Fachbereich Erziehungswissenschaften und Psychologie, Klinische Psychologie und Psychotherapie, Freie Universität Berlin, Berlin, Deutschland

**Keywords:** Zugang Versorgung, Gatekeeper, Lotsenfunktion, Screening, Weiterleitung, Access to care, Gatekeeping, Pilot function, Screening, Referral

## Abstract

**Hintergrund:**

Die postpartale Depression (PPD) zählt zu den häufigsten psychischen Erkrankungen in der Postpartalzeit. Unbehandelt kann sie teils folgenschwere Auswirkungen auf die Mutter-Kind-Beziehung und die Entwicklung des Kindes haben. Um mögliche negative Auswirkungen verhindern zu können, sind eine frühzeitige Diagnostik betroffener Mütter und eine professionelle Betreuung essenziell.

**Ziel der Arbeit:**

Der vorliegende Artikel exploriert das Zuständigkeitsgefühl der 4 Primärversorger:innen in der Postpartalzeit: Hebammen, Gynäkolog:innen, Hausärzt:innen und Pädiater:innen, und untersucht den Umgang mit der Erkrankung sowie die Barrieren und Optimierungsmöglichkeiten in der Versorgung.

**Material und Methoden:**

Die primären Versorger:innen von Frauen nach einer Geburt in Deutschland wurden in 4 voneinander unabhängigen Studien befragt. Mit Hebammen, Gynäkolog:innen und Hausärzt:innen wurden quantitative Befragungen mittels Fragebögen durchgeführt, mit Vertreter:innen des deutschen Berufsverbands der Kinder- und Jugendärzte e. V. (BVKJ) eine qualitative Telefoninterviewbefragung. Es erfolgte eine systematische vergleichende Analyse.

**Ergebnisse und Diskussion:**

Hebammen und Gynäkolog:innen zeigten ein deutlich höheres Zuständigkeitsgefühl für das Erkennen und die Therapie der PPD als Hausärzt:innen und Pädiater:innen. Als zentrale Voraussetzung für eine Verbesserung der Versorgungssituation in Deutschland wurden von allen 4 Berufsgruppen eine engere interdisziplinäre Zusammenarbeit und somit ein größeres Angebot an Überweisungs- und Therapiemöglichkeiten genannt. Auch eine einheitliche Regelung der finanziellen Vergütung ist für alle Versorger ein wichtiger Aspekt.

**Zusatzmaterial online:**

Zusätzliche Informationen sind in der Online-Version dieses Artikels (10.1007/s00103-022-03545-8) enthalten.

## Abstract (en)

**Background:**

Postpartum depression (PPD) is one of the most common mental illnesses in the postpartum period. If left untreated, it can have serious consequences for the mother–child relationship and the development of the child. In order to prevent possible negative effects, early diagnosis of affected mothers and professional care are essential.

**Aim of the study:**

This article explores the sense of responsibility of the four primary care providers in the postpartum period—midwives, gynaecologists, general practitioners and paediatricians—and examines how they deal with the disease as well as the barriers and possibilities for optimisation in care.

**Materials and methods:**

The primary care providers of postpartum women in Germany were interviewed in four independent studies. Quantitative questionnaires were used to interview midwives, gynaecologists and general practitioners, and a qualitative telephone survey was conducted with representatives of the German Association of Paediatricians and Adolescents (BVKJ). A systematic comparative analysis was carried out.

**Results and discussion:**

Midwives and gynaecologists showed a significantly higher sense of responsibility for the recognition and treatment of PPD than general practitioners and paediatricians. Closer interdisciplinary cooperation and thus a wider range of referral and therapy options were named by all four professional groups as a central prerequisite for improving the care situation in Germany. A uniform regulation of financial remuneration is also an important aspect for all providers.

## Hintergrund

Die postpartale Depression (PPD) zählt zu den häufigsten Komplikationen nach der Geburt eines Kindes [1]. Etwa 10–15 % der Wöchnerinnen^1^ entwickeln in dieser Zeit eine klinisch relevante Depression mit multifaktorieller Genese [2]. Auch Väter können in bis zu 10 % der Fälle von einer postpartalen Depression betroffen sein [3]. Psychische Vorerkrankungen wie Depressionen und Angststörungen, psychosoziale Stressoren in der Schwangerschaft oder auch traumatische Geburtserlebnisse zählen zu den häufigsten Risikofaktoren für postpartale Depression [4]. Die Klassifikation der PPD erfolgt nach den internationalen Klassifikationssystemen International Statistical Classification of Diseases and Related Health Problems (ICD-10) und Diagnostic and Statistical Manual of Mental Disorders (DSM-5) analog zu der Einordnung depressiver Störungen in anderen Lebensphasen [2, 5, 6]. Zu den Symptomen einer PPD gehören depressive Verstimmungen, verminderter Antrieb, Interessenverlust, Appetitlosigkeit, Schlaflosigkeit und unter Umständen suizidale Gedanken [7]. Zudem können auf das Muttersein bezogenes Insuffizienzerleben sowie Zwangsgedanken, dem Kind etwas anzutun, auftreten [2].

Differenzialdiagnostisch abzugrenzen ist die PPD von der postpartalen Dysphorie, auch Babyblues genannt, und der postpartalen Psychose. Während die postpartale Dysphorie meist in den ersten Tagen nach der Entbindung auftritt, kann sich eine PPD innerhalb des gesamten ersten Jahres nach der Geburt manifestieren [8]. Diese zeitliche Einteilung hat sich sowohl aus klinischen Gesichtspunkten als auch im Rahmen wissenschaftlicher Betrachtungen der PPD durchgesetzt [9], wenngleich das DSM‑5 einen Symptombeginn antepartal bis 4 Wochen nach der Geburt voraussetzt [6]. Die postpartale Psychose ist eine schwerwiegende, aber mit einer geschätzten Prävalenz von 0,1–0,2 % eine seltene Erkrankung [7, 10].

Eine postpartale Depression kann schwerwiegende Folgen für die ganze Familie haben, denn insbesondere eine maternale Depressivität führt häufig zu einer gestörten Interaktion zwischen Mutter und Kind [11, 12]. Durch Beeinträchtigung des mütterlichen Empathievermögens, der Fürsorge und Erziehungskompetenz sowie Beziehungsgestaltung [13] kann sich beim Kind eine erhöhte Irritabilität mit schwächer ausgeprägten Fähigkeiten zur Selbstregulation einstellen und die Entwicklung einer unsicheren Bindung beobachtet werden [10]. Diese kann sich wiederum negativ auf die physische, sprachliche, kognitive und emotionale Entwicklung des Kindes auswirken [12, 14, 15]. Eine unbehandelte PPD weist ein hohes Risiko für eine Chronifizierung auf [16] und kann in seltenen Fällen zum Suizid oder Infantizid führen [9, 17]. Nicht zuletzt wurde zudem beobachtet, dass das Vorliegen einer PPD des einen Elternteils zur Entwicklung bzw. zur Verschlechterung von Symptomen (Aggravation) beim zweiten Elternteil führen kann [18].

Das breite Spektrum postpartal psychischer Störungen und die Schwierigkeit, diese von normalen Anpassungsvorgängen nach der Geburt abzugrenzen, erschweren neben psychosozialen Faktoren die Erkennung einer PPD [2]. Vor dem Hintergrund der hohen Prävalenzrate und der weitreichenden möglichen Folgen sind eine frühzeitige Erkennung und Therapieanbindung betroffener Frauen von entscheidender Bedeutung [19–21]. Allerdings hat sich in Deutschland bislang kein flächendeckendes Routinescreening etabliert [9], weshalb PPD oftmals unentdeckt und unbehandelt bleiben. Zudem wird die rechtzeitige Inanspruchnahme von Hilfsangeboten durch unzureichendes Wissen sowie Schuld- und Schamgefühle seitens der Betroffenen erschwert [10].

Die nationale AWMF-S3-Leitlinie der Arbeitsgemeinschaft der Wissenschaftlichen Medizinischen Fachgesellschaften e. V. zur unipolaren Depression enthält Empfehlungen zur Diagnostik und Behandlung der PPD für alle Versorger:innen [9]. Das am häufigsten eingesetzte Screeninginstrument ist die Edinburgh Postnatal Depression Scale (EPDS; [9]). Ein Scoring-Wert von ≥ 10 gibt erste Hinweise auf das Vorliegen einer PPD. Zur weiteren Abklärung der Verdachtsdiagnose sollte stets ein klinisches Interview erfolgen [22, 23]. Für die Behandlung von PPD gibt es eine Vielzahl an Therapieoptionen: Neben der psychopharmakologischen Behandlung gilt die Psychotherapie – speziell die interpersonelle Therapie (IPT) und die kognitive Verhaltenstherapie (KVT) – als besonders wirksam [9]. Sofern eine stationäre Behandlung erforderlich ist, sollte diese falls möglich in einer Klinik mit einer Mutter-Kind-Einheit erfolgen, um eine Trennung von Mutter und Kind zu vermeiden [10]. Als niedrigschwellige Anlaufstellen können zudem Beratungsstellen und Selbsthilfegruppen dienen [2].

**Figure 1:**
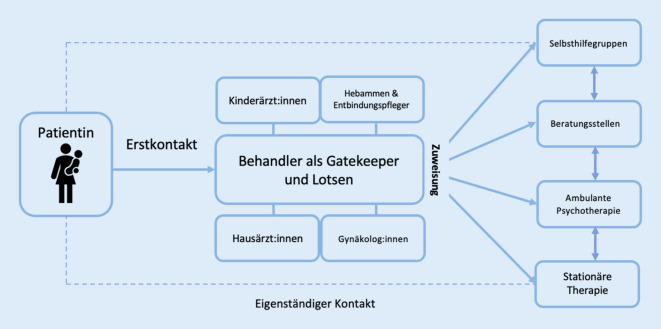

Erfreulicherweise sind Frauen in Deutschland meist während und nach der Schwangerschaft gut ins Gesundheitssystem eingebunden und stehen in Kontakt zu Hebammen/Entbindungspflegern (im Folgenden vereinfachend Hebammen genannt), Gynäkolog:innen, Pädiater:innen sowie Hausärzt:innen [24, 25]. Diese nehmen somit auch eine wichtige Rolle in der Diagnostik von psychischen Erkrankungen in der Perinatalzeit^2^ ein. Abb. 1 stellt die verschiedenen Zugangswege zur Versorgung (Pathways to Care) für eine postpartal depressive Betroffene dar.

In der frühen Postpartalzeit werden Wöchnerinnen durch Hebammen in physischen und psychischen Belangen betreut und beraten, wobei der Schwerpunkt in erster Linie auf der Förderung der körperlichen Regeneration liegt [22]. Nach 6–8 Wochen sollen Frauen für einen Nachsorgetermin in der gynäkologischen Praxis vorstellig werden [26]. Zu Kinderärzt:innen besteht ein frühzeitiger und ab dann engmaschiger Kontakt im Rahmen der pädiatrischen Früherkennungsuntersuchungen [27]. Hausärzt:innen sind niedrigschwellige, erste Ansprechpartner:innen bei jeglichen gesundheitlichen Beschwerden [28] und sind darüber hinaus insbesondere in ländlichen Gebieten für die Durchführung von U -Untersuchungen zuständig [29]. Damit bieten sich diesen Zuweiser:innen zahlreiche Gelegenheiten, um die Symptome einer PPD frühzeitig zu erkennen und gegebenenfalls weitere Maßnahmen einzuleiten bzw. an Therapeut:innen weiterzuleiten [30, 31].

Die in der Literatur häufig mit der hausarztzentrierten Versorgung assoziierte Lotsen- und Zuweiserfunktion (engl.: „gatekeeping“) geht in der Praxis längst über eine reine Beratungsfunktion hinaus und verfolgt den Zweck der Koordination und Steuerung der ambulanten Gesundheitsversorgung. Demnach können auf diesem Weg auch Hebammen, Gynäkolog:innen und Kinderärzt:innen (Abb. 1) eine wichtige Lotsenfunktion einnehmen, da diese ähnlich wie Hausärzt:innen sachverständig, vertrauenswürdig und im Versorgungsprozess involviert sind. Je nach Bedarf können Zuweiser:innen also die Versorgung der Patient:innen innerhalb eines Versorgungsbereichs koordinieren und mitbestimmen, sektorenübergreifend und begleitend im Behandlungsverlauf zur Seite stehen [32] und durch eine koordinierte Versorgung eine Steigerung der Behandlungsqualität erzielen [33].

Nicht nur in Deutschland wird die Versorgungssituation der postpartal depressiven Frauen als unzureichend bewertet [13, 19, 21, 34]. Zahlreiche nationale und internationale Expert:innen vertreten die Meinung, dass eine frühzeitige Identifikation und zeitnahe Behandlung einer PPD oftmals nicht gewährleistet ist [7, 13, 20, 35] und diese damit ein ernstzunehmendes öffentliches Gesundheitsproblem darstellt [8]. Insbesondere im angloamerikanischen Sprachraum sind PPD bereits seit Längerem in den Fokus zahlreicher Fachkreise und der Öffentlichkeit gerückt [30].

Eine erhebliche Diskrepanz zwischen einem zwar oftmals stark ausgeprägten Verantwortungsgefühl und dafür vergleichsweise niedrigen Screening‑, Weitervermittlungs- bzw. Behandlungsraten wurde bereits in mehreren Studien für alle Versorgenden berichtet [35]. Bei einer Befragung wurde unter US-amerikanischen Gynäkolog:innen mit 87 % [36] ein stark ausgeprägtes Verantwortungsgefühl für die Erkennung der PPD beobachtet. In der Befragung von Leddy et al. (2011) screenten fast 3 Viertel der Gynäkolog:innen ihre Patientinnen post partum systematisch auf das Vorliegen einer PPD. In einer anderen US-amerikanischen Studie mit einer gemischten Stichprobe aus Hausärzt:innen und Gynäkolog:innen betrug das Verantwortungsgefühl 97 % [37]. Bei einer israelischen Befragung fühlten sich sogar 100 % der Hausärzt:innen für die Erkennung der PPD verantwortlich und äußerten im Vergleich zu den Kinderärzt:innen (65 %) wesentlich häufiger (91 %) die Bereitschaft, ein Screeninginstrument in ihrer Praxis zu etablieren [38]. Nach einem Review von Goldin Evans et al. (2015) setzen Kinderärzt:innen (7 %) insgesamt im Vergleich zu den Hausärzt:innen (31 %) und Gynäkolog:innen (37 %) international wesentlich seltener validierte Instrumente für das Screening auf PPD ein.

Eine Analyse des Umgangs der verschiedenen Berufsgruppen mit der Identifikation und Versorgung von postpartal depressiven Frauen steht für das deutsche Gesundheitssystem noch aus. Im Rahmen von in sich geschlossenen und unabhängigen Studien wurden im Rahmen der vorliegenden Arbeit Befragungen der zentralen Berufsgruppen (Hebammen, Gynäkolog:innen, Hausärzt:innen sowie Kinder- und Jugendärzt:innen) durchgeführt. Folgende Fragestellungen wurden in 4 Einzelstudien untersucht und werden vergleichend dargestellt:

1. Inwiefern fühlen sich die Versorger:innen für die Identifikation und Versorgung der PPD in Deutschland zuständig (Zuständigkeit)?
2. Was tun Versorger:innen zur Identifikation und bei Vorliegen einer PPD (Umgang)?
3. Welche Barrieren und Optimierungsmöglichkeiten sehen die Versorger:innen in der Zuweisung und Versorgung von Frauen mit PPD (Barrieren)?

## Material und Methoden

Unsere Forschungsgruppe hat 4 Einzelstudien durchgeführt, um die Sichtweise der Primärversorger:innen auf die PPD und die aktuelle Versorgungssituation betroffener Mütter zu erfassen. Methoden und Material der Einzelstudien unterscheiden sich, sodass zur Beantwortung der Fragestellungen sowohl quantitative Daten von Fragebogenerhebungen von 1) Gynäkolog:innen [39], 2) Hebammen [40] und 3) Hausärzt:innen [41] sowie 4) qualitative Daten von Telefoninterviews mit den Landesvertreter:innen des Berufsverbands der Kinder- und Jugendärzt:innen (BVKJ; [42]) herangezogen wurden.

### Stichprobenbeschreibung und Material Gynäkolog:innen, Hebammen, Hausärzt:innen

1034 niedergelassene Gynäkolog:innen wurden im Rahmen einer bundesweiten Fragebogenerhebung zu ihrem Umgang mit Patientinnen mit Verdacht auf PPD befragt. Die Stichprobe wurde zufällig aus einer Grundgesamtheit von *N* = 9823 Adressen niedergelassener Gynäkolog:innen in Deutschland ausgewählt, die in der Datenbank des Adressdienstleisters „MediAdressen Select“ erfasst waren (Rücklaufquote 35 %).

165 Hebammen wurden über die Hebammenverbände Schleswig-Holstein, Niedersachsen und Hamburg rekrutiert (Rücklaufquote: 8 %) und nahmen an einer Onlinebefragung über die Web-Applikation SoSci Survey teil.

217 Hausärzt:innen wurden per E‑Mail (über einen Adressdatensatz des Dienstleisters „SciTrace“ sowie über eine manuelle Recherche der Kontaktdaten) rekrutiert und nahmen ebenfalls über SoSci Survey an einer Onlinebefragung teil (Rücklaufquote: 7 %).

Die Auswertung der Daten erfolgte deskriptiv und inferenzstatistisch mittels SPSS. Tab. 1 gibt einen Überblick über die Stichprobe der quantitativen Surveys inklusive Rekrutierungszahlen und Soziodemografie.

**Table 1:** 

	Quantitative Surveys					
	Gynäkolog:innen		Hebammen		Hausärzt:innen	
Rekrutierung (*N*)	3000		2051		3300	
Teilnehmer:innen (*n*)	**1034**^**a**^		**165**^**a**^		**217**^**a**^	
Nicht-Teilnehmer:innen (*n*)	1966		1866		3083	
Rücklaufquote (%)	**35**		**8**		**7**	
	***N***	**%**	***N***	**%**	***N***	**%**
**Geschlecht**	**(*n*** **=** **1022**^b^**)**		**(*n*** **=** **159**^b^**)**		**(*n*** **=** **193**^b^**)**	
Weiblich	656	64	159	100	106	55
Männlich	366	36	k. A.	k. A.	87	45
**Alter**						
Mittelwert	52		46		50–60^c^	
(Spannweite)	(35–73)		(24–72)		(30–> 60)^c^	
**Berufstätigkeit in Jahren**						
Mittelwert	24		21		24	
(Spannweite)	(1–55)		(1–48)		(3–47)	
**Einzugsgebiet**	**(*n*** **=** **1003**^b^**)**		**(*n*** **=** **160**^b^**)**		**(*n*** **=** **192**^b^**)**	
Eher ländlich	385	38	56	35	81	42
Eher städtisch	251	25	53	33	62	32
Ländlich und städtisch	367	37	51	32	49	26
**Mind. 1 erworbene Zusatzqualifikation**	**(*n*** **=** **621**^**d**^**)**				**(*n*** **=** **157**^**d**^**)**	
Psychosomatische oder psychotherapeutische Zusatzqualifikation	448	74	n. u.	n. u.	68	43

*k. A.* keine Angaben, *n. u.* nicht untersucht

^a^ *N* entspricht der Gesamtheit aller Fragebogenrückläufer, auch unvollständig ausgefüllte Fragebögen wurden gezählt

^b^ Abweichendes *N* entspricht den Angaben ohne Missings

^c^ Das Alter der Hausärzt:innnen wurde kategorial in Altersklassen von 10 Jahren erhoben sowie < 30 J. und > 60 J. und anstelle des Mittelwertes wird hier die mit 42 % überwiegende Altersgruppe angegeben

^d^ Abweichendes *N* entspricht ausschließlich Positivantworten, Missings und „keine Zusatzqualifikation“ wurden nicht gezählt

Für die Befragung der niedergelassenen Gynäkolog:innen wurde ein 28 Items umfassender Fragebogen verwendet, der auf der Grundlage von Ergebnissen aus Befragungen vergleichbarer Stichproben [43] entwickelt wurde. Für die Onlinebefragung der Hebammen und Hausärzt:innen wurde der Fragebogen an die Berufsgruppen sprachlich angepasst und 15 Items (Hebammenbefragung) und 21 Items (Hausärzt:innen-Befragung) umgesetzt. Alle Fragebögen enthielten in offenen und geschlossenen Antwortformaten Angaben zu a) Praxis und Person, b) Diagnostik und Umgang mit Patientinnen mit Verdacht auf PPD sowie c) system- und patientenbezogenen Barrieren im Management der Identifikation und Überleitung in Hilfen. Bei mehreren Items waren Mehrfachnennungen möglich. Einschätzungen wurden entweder dichotom („Ja“, „Nein“) oder in einer 4‑ bis 5‑stufigen Likert-Skala vorgegeben. Die Tabelle im zusätzlichen Onlinematerial stellt die Items und die dazugehörigen Antwortskalen aus den 3 Fragebögen dar.

### Stichprobenbeschreibung und Material Kinder- und Jugendärzt:innen

Die Erhebung der Daten bei den Pädiater:innen erfolgte durch strukturierte Interviews (*N* = 17) mit Vertreter:innen aus allen 17 Landesverbänden des Berufsverbands der Kinder- und Jugendärzte e. V. (BVKJ). Die Befragung erfolgte mittels Interviews, um die Relevanz des Themas in der Berufsgruppe explorativ zu ermitteln, da in der Pädiatrie elterliche Erkrankungen nicht primär zur Versorgung gehören.

Die ca. 15-minütigen Interviews wurden per Telefon durchgeführt, aufgezeichnet und im Anschluss transkribiert. Zur Durchführung der Interviews wurde ein Leitfaden entwickelt, der Fragen zu a) Praxis, Schwerpunkt und Person, b) Diagnostik und Umgang mit Patientinnen mit Verdacht auf PPD und c) Voraussetzungen, die geschaffen werden müssten, enthielt. Die Daten wurden nach der inhaltlich strukturierten qualitativen Inhaltsanalyse nach Kuckartz [44] ausgewertet.

Im Durchschnitt arbeiten die Befragten (*N* = 17) seit 27 Jahren als niedergelassene Kinder- und Jugendärzt:innen und vertreten für den BVKJ die Interessen ihres jeweiligen Bundeslandes. Das Einzugsgebiet ist von 59 % der Befragten städtisch, bei 6 % eher ländlich und bei 35 % sowohl städtisch als auch ländlich. Etwas weniger als die Hälfte (41 %) setzen in ihrer eigenen Praxis Screenings zur postpartalen Depression bei Müttern ein.

## Ergebnisse

### Befragung von Gynäkolog:innen, Hebammen und Hausärzt:innen

Die wichtigsten Ergebnisse der Befragungen von Gynäkolog:innen, Hebammen und Hausärzt:innen sind in Tab. 2 vergleichend dargestellt.

**Table 2:** 

	Berufsgruppen		
	Hebammen (%)	Gynäkolog:innen (%)	Hausärzt:innen (%)
*Zuständigkeitsgefühl PPD*			
Auf jeden Fall/eher zuständig	95	96	80
*Erfassung von PPD-Symptomen*			
Bei allen postpartalen Frauen	98	k. A.	17
*Identifikation PPD durch* (Mehrfachnennungen möglich)			
Gespräch	89	98	93
Fragebögen	41	17	6
Systematische Fragebogenerhebung/Routine	12	6	k. A.
*Umgang mit PPD *(Mehrfachnennungen möglich)			
Beratungsgespräch	74	84	63
Vermittlung an Beratungsstelle	50	87	40
Vermittlung an (Psycho‑)Therapeut:in/Klinik	48	k. A.	64
Vermittlung an Ärzt:innen	55	k. A.	18
Verschreibung Antidepressiva	k. A.	k. A.	16
*Systembezogene Barrieren in der Versorgung der PPD *(Mehrfachnennungen möglich)			
Fehlen effektiver Behandlungsmöglichkeiten	59	50	43
Begrenzte Beratungszeit	54	74	42
Geringe Vergütung bzw. fehlende Erstattung durch die Krankenkassen	46	53	k. A.
Qualität der/Wartezeiten zur Überweisungsmöglichkeit	53	k. A.	87
*Patientenbezogene Barrieren in der Versorgung der PPD *(Mehrfachnennungen möglich)			
Nichtinanspruchnahme durch die Mutter	66	45	31
Fehlende Diagnoseakzeptanz durch die Mutter	41	48	42
Angst vor Stigmatisierung	k. A.	k. A.	48

*k.* *A.* keine Angaben

#### Zuständigkeit zur Identifikation der PPD

Nach ihrem persönlichen Zuständigkeitsgefühl für die Detektion und Intervention bei PPD befragt, gaben alle 3 Berufsgruppen ein hohes Zuständigkeitsgefühl an. Die Hebammen stimmten zu 95 % und die befragten Gynäkolog:innen zu 96 % mit „ja, auf jeden Fall zuständig“ sowie „ja, eher zuständig“. Aus der Gruppe der Hausärzt:innen gaben ca. 80 % der Teilnehmer:innen an, sich für die Erkennung der PPD zuständig zu fühlen.

#### Erfassung der postpartalen Depression

Bei der Erfassung der PPD wurden die Teilnehmenden nach den von ihnen genutzten Erhebungsinstrumenten gefragt, wobei Mehrfachnennungen möglich waren. 98 % der Hebammen gaben an, Anzeichen einer PPD bei ihren Patientinnen zu erheben. Dies geschah zu 89 % im Patientengespräch, wobei ca. 75 % gezielt Anzeichen erfragten. 41 % verwendeten einen Fragebogen, wobei 12 % einen routinemäßigen und 38 % einen Gebrauch im Bedarfsfall angaben. 53 % gingen zudem Hinweisen durch Dritte nach.

Die Gynäkolog:innen gaben zu 98 % an, Anzeichen einer PPD im Patientengespräch zu erheben, in 69 % durch aktives Erfragen. 17 % nutzten einen Fragebogen, in 6 % auch routinemäßig. Mehr als die Hälfte der Befragten nutzte mehr als 1 Screeninginstrument.

Von den befragten Hausärzt:innen gaben 97 % an, Anzeichen einer PPD bei ihren Patientinnen zu erfassen. Dies geschah zu 93 % im Gespräch. Lediglich 6 % gaben an, einen Fragebogen zu nutzen. Circa 17 % der Hausärzt:innen erheben routinemäßig Anzeichen einer PPD bei jeder Patientin nach der Geburt. Hausärzt:innen verwiesen zu 64 % an eine/n Psychotherapeut:in, zu 60 % an ein psychosoziales Unterstützungsangebot, wie bspw. eine Familienhebamme oder Haushaltshilfe, sowie zu 40 % an eine Beratungsstelle. 16 % der Hausärzt:innen gaben zudem an, Antidepressiva zu verordnen.

#### Umgang mit der PPD

Als primäre Interventionsmöglichkeit gaben sowohl Hebammen (74 %) als auch Gynäkolog:innen (84 %) und Hausärzt:innen (63 %) an, das Gespräch mit der Betroffenen zu suchen. Die befragten Hebammen vermittelten die Mütter zudem in 50 % an eine Beratungsstelle weiter oder überwiesen an eine/n Gynäkolog:in (55 %) oder Psychotherapeut:in (48 %). In der Stichprobe der Gynäkolog:innen wird zu 87 % an eine Beratungsstelle vermittelt und ähnlich häufig eine Überweisung an eine/n Psychotherapeut:in ausgestellt. Je 1/3 der Befragten gab eine Vermittlung an eine (Familien‑)Hebamme sowie Strukturen der Frühen Hilfen an.

#### Barrieren und Optimierungsmöglichkeiten der PPD-Versorgung

Hindernisse und Einflussfaktoren in der Zuweisung sowie in der Behandlung der PPD sahen 59 % der Hebammen im Fehlen effektiver Behandlungsmöglichkeiten und 53 % in wenig qualitativen Überweisungsmöglichkeiten. Weitere 54 % nannten die geringe verfügbare Beratungszeit der Behandler:innen, rund 46 % die geringe Vergütung bzw. fehlende Erstattung durch die Krankenkasse als Hindernis. Viele Hebammen sahen zudem das Problem der Ablehnung des Interventionsangebots (66 %) oder eine fehlende Diagnoseakzeptanz (41 %) durch die Mütter.

Bei den Gynäkolog:innen wurde am häufigsten (74 %) die begrenzte Beratungszeit sowie von ca. der Hälfte die geringe Vergütung bzw. fehlende Erstattung als Einflussfaktor für die Versorgung bei PPD benannt. Ebenfalls ca. die Hälfte gab das Fehlen effektiver Behandlungsmöglichkeiten an. Eine fehlende Diagnoseakzeptanz und Ablehnung durch betroffene Mütter wurden auch hier mit je 48 % und 45 % benannt.

Die Gruppe der Hausärzt:innen gab zu 87 % die lange Wartezeit auf einen Therapieplatz an, gefolgt von fehlenden effektiven Behandlungs‑/Überweisungsmöglichkeiten (43 %) sowie einer begrenzten Beratungszeit (42 %). Auch eine fehlende interdisziplinäre Zusammenarbeit wird von 42 % bemängelt. Knapp die Hälfte der Befragten gab ebenfalls eine fehlende Diagnoseakzeptanz (42 %) sowie Nichtinanspruchnahme (31 %) durch die Mütter an.

94 % der befragten Hebammen gaben einen deutlichen Optimierungsbedarf der aktuellen Versorgungssituation in Deutschland an. Vornehmlich wurde Potenzial in den Bereichen der interdisziplinären Zusammenarbeit (87 %) und zertifizierten Fortbildungen (80 %) gesehen. 60 % gaben zudem eine Neuregelung der Erstattung der Beratung an. Für 60 % der befragten Hebammen besteht zudem Optimierungspotenzial in der systematischen Erfassung psychosozialer, finanzieller sowie psychischer Belastungen, bspw. auch mittels validierter Fragebögen.

Bei den Gynäkolog:innen wurde mit 71 % vor allem eine Verbesserung der Zusammenarbeit und eine Neuregelung der Beratungserstattung mit 56 % angegeben. Auch sie sahen eine verbesserte Diagnostik (43 %) und die Teilnahme an zertifizierten Fortbildungen (33 %) als mögliche Optimierungsoption.

Durch die Hausärzt:innen wurde der Wunsch nach qualifizierten Fortbildungen sowie ein Verbesserungsbedarf in der interdisziplinären Zusammenarbeit geäußert. Sie gaben zu 46 % an, über mehr Wissen zur PPD verfügen zu wollen. Die Leitlinie sei nur ca. 20 % der Befragten bekannt gewesen.

### Ergebnisse der Befragung von Vertreter:innen der Landesverbände der Kinder- und Jugendärzt:innen

In Tab. 3 werden die wichtigsten Ergebnisse der telefonischen Interviews mit den Vertreter:innen der Landesverbände der Kinder- und Jugendärzt:innen dargestellt. Diese werden im Folgenden in Bezug zu den schriftlichen Aussagen der Gynäkolog:innen, Hebammen und Hausärzt:innen gesetzt.

**Table 3:** 

	Vertreter:innen des BVKJ (*N* = 17)
**Zuständigkeitsgefühl PPD**a	
**–**	Ca. 58 % *kein* Zuständigkeitsgefühl
**Wahrnehmung und Umgang mit PPD (Mehrfachnennungen möglich)**	
–	47 % Bekanntheit Screeningverfahren
	29 % Fachwissen zu PPD
	6 % Routinescreening zu PPD
**Intervention bei PPD (Mehrfachnennungen möglich)**	
**–**	53 % Aktendokumentation
	47 % ausführliches Beratungsgespräch
	47 % Vermittlung (Psycho‑)Therapeut:in/Klinik
	29 % Vermittlung Fachärzt:in
	29 % Vermittlung an andere Versorger
	18 % Informationsmaterial
**Einflussfaktoren/Hindernisse in der Versorgung der PPD (Mehrfachnennungen möglich)**	
**–**	*Barrieren Ärzt:innen:*
	59 % geringes Zuständigkeitsgefühl
	18 % Bereitschaft zur Auseinandersetzung mit der Mutter
	35 % zeitlicher Rahmen
	*Hemmnisse Eltern:*
	12 % Sorge vor rechtlichen Konsequenzen
	29 % soziale Erwünschtheit als Screeningbias
	12 % Sprachbarrieren
**Mögliche Optimierung der PPD-Versorgung (Mehrfachnennungen möglich)**	
**–**	47 % Fortbildungen
	29 % evaluiertes Screeninginstrument
	47 % Praktikabilität des Screenings
	29 % Kostenübernahme
	24 % Weiterleitungsmöglichkeiten
	18 % Interdisziplinäre Versorgung

Laut den Aussagen der befragten Landesvertreter:innen wird in nur einem Bundesland ein Screening zur Erfassung einer PPD genutzt. 47 % gehen aber davon aus, dass Pädiater:innen zumindest ein Screeningverfahren bei Verdacht auf eine postpartale Depression bekannt ist. Nach dem Fachwissen befragt, gaben 71 % an, dass Kinder- und Jugendmediziner:innen nicht ausreichend über das Krankheitsbild informiert sind. Ein generelles Screening zu PPD bei Kinder- und Jugendärzt:innen wurde allerdings mehrheitlich – unter bestimmten Voraussetzungen – befürwortet.

Als primäre Intervention wurde von 53 % ein Vermerk in der Patientenakte angegeben. In der Befragung nannten 47 % ein ausführliches Beratungsgespräch als Maßnahme bei Verdacht auf eine PPD. Je ca. die Hälfte der Befragten nannte die Vermittlung an eine Beratungsstelle, eine/n Psychotherapeut:in oder eine/n Fachärzt:in (29 %) als mögliche Intervention. Zudem gaben 18 % an, Informationsmaterial auszuhändigen.

Als hinderlich für die Versorgung wurde eine häufig fehlende Bereitschaft der Kinder- und Jugendärzt:innen zur Auseinandersetzung mit den psychischen Belastungen der Mutter angesehen, zudem mangele es im Rahmen der kinderärztlichen Konsultation oft an Zeit. Mehr als der Hälfte der Befragten erschien ein Screening im Rahmen der U3-Untersuchung unter bestimmten Voraussetzungen allerdings als sinnvoll. Als Voraussetzung wurde von einem Großteil der Befragten eine spezifische Fortbildung (47 %) sowie von 29 % ein evaluiertes Screeningtool gewünscht. Zudem müssten die Praktikabilität (47 %) sowie eine angemessene Vergütung (29 %) gewährleistet sein.

## Diskussion

Untersucht wurde, welche Rollen die Versorger:innen von Frauen nach der Geburt (Hebammen, Gynäkolog:innen, Kinder- und Jugendärzt:innen sowie Hausärzt:innen) in der Früherkennung und Versorgung der PPD einnehmen.

Trotz eines engen Kontakts von Behandler:innen in der frühen Postpartalzeit, Screeninginstrumente wie dem EPDS sowie pharmakologischer und psychotherapeutischer Therapiemöglichkeiten werden die Früherkennung und Weiterleitung bzw. Behandlung der PPD noch immer als unzureichend bewertet [13, 19, 21, 34]. Da es sich bei der PPD um eine ernstzunehmende psychische Erkrankung von Müttern handelt, die sich nachteilig auf die Entwicklung der Kinder auswirken und schwerwiegende Folgen für die Familien haben kann, sind eine frühe Diagnostik, Aufklärung und Weiterleitung zu bestehenden Unterstützungs- und Behandlungsangeboten essenziell. Idealerweise haben die 4 wichtigsten Versorger:innen einen optimalen Zugang zu Schwangeren und jungen Müttern mit ihren Kindern, um neben der somatischen Behandlung psychische Auffälligkeiten oder Krankheitsanzeichen und das daraus resultierende Risiko für das Kind zu erkennen.

Unsere Studienergebnisse zeigen, dass die Zuständigkeit für das Krankheitsbild der PPD in Deutschland im direkten Vergleich der Berufsgruppen vor allem durch die Gynäkolog:innen und Hebammen gesehen wird. Die befragten Hausärzt:innen fühlten sich weniger, die Vertreter:innen der BVKJ mit 42 % am wenigsten für das Krankheitsbild zuständig. Damit stehen die Studienergebnisse im Einklang mit den internationalen Vergleichen.

Obwohl bereits gut validierte Erhebungsinstrumente wie der EPDS vorhanden sind, lässt sich im Rahmen unserer Untersuchung eine große Diskrepanz zwischen den vorhandenen Möglichkeiten der Diagnostik und der tatsächlichen Erhebung in der Praxis erkennen. Ein routinemäßiger, systematischer Einsatz eines Fragebogens, wie bspw. des EPDS, wurde professionsübergreifend nur von einem geringen Anteil der Versorger:innen angegeben (6–12 %). Der EPDS-Bogen ermöglicht ein einfaches und validiertes Screening der Symptome der PPD (siehe Empfehlung in der AWMF-Leitlinie) und steht kostenfrei in mehreren Sprachen zur Verfügung. Dennoch wünschten sich alle Berufsgruppen eine verbesserte Diagnostik im Sinne einer systematisierten Erfassung der Symptome der PPD. Dies lässt auf ein teilweise unzureichendes Fachwissen der Versorger:innen über PPD und deren Screening schließen, welchem mit spezifischen Fortbildungen begegnet werden sollte. In Übereinstimmung damit äußerten alle befragten Berufsgruppen ein großes Interesse an zertifizierten Fortbildungen und sahen diese als wichtige Optimierungsmöglichkeit in der PPD-Versorgung. Kurze Onlineweiterbildungskurse könnten hier eine Möglichkeit darstellen, um Versorger:innen über Anzeichen einer PPD und die Nutzung des EPDS-Bogens aufzuklären. In diesen könnte auch auf internetbasierte Screeningtools und Aufklärungsangebote (z. B.: www.smart-moms.de) hingewiesen werden. Diese könnten das Screening für Versorger:innen noch praktikabler und zeitsparender gestalten. Das Fundament für ein adäquates Management der PPD sollte in einem routinemäßigen Screening von Frauen nach der Geburt durch Primärversorger:innen liegen. So könnten Gynäkolog:innen den EPDS-Bogen bei allen Wöchnerinnen im Rahmen der Nachsorgetermine [45] und Pädiater:innen im Rahmen der U‑Untersuchungen einsetzen [42, 46], um Betroffene im Anschluss an weitergehende qualifizierte Versorgung weiterzuleiten.

Als Barrieren wurden von Hebammen und Gynäkolog:innen vor allem die begrenzte Beratungszeit der Behandler:innen sowie das Fehlen einer angemessenen monetären Vergütung der Beratung genannt: Im Rahmen der U‑Untersuchungen sehen sich die Kinder- und Jugendärzt:innen einem Zeitmangel gegenüber. Die Betreuung einer postpartalen Frau durch Hebammen oder Geburtshelfer wird lediglich bis 8 Wochen nach Entbindung vergütet, solang keine ärztliche Anordnung vorliegt [48], und in Deutschland tätige Hebammen betreuen im Schnitt doppelt so viele Geburten wie ihre Kolleg:innen in europäischen Nachbarländern [47]. Für die ärztlich tätigen Berufsgruppen gibt es keine spezifische Abrechnungsziffer, mit welcher das Screening auf PPD oder eine intensivierte Beratung einer Mutter mit PPD vergütet werden könnte [49]. Eine Neuregelung der Beratungs- und Versorgungskosten der PPD könnte somit einen Anreiz zur Optimierung der Versorgung darstellen. Ein weiterer Ansatz für eine optimierte Vergütung liegt in einer Anpassung der Diagnosekataloge ICD-10 und DSM‑5.

Die Optimierungsmöglichkeit, die von Hebammen sowie Gynäkolog:innen am häufigsten genannt wurde, ist eine engere Zusammenarbeit der Versorger:innen, um der Problematik fehlender Behandlungs- und Überweisungsmöglichkeiten und langer Wartezeiten auf einen Therapieplatz zu begegnen. Aus der Untersuchung wird der klare Bedarf abgeleitet, dass zur Überweisung auch geeignete Weiterleitungsmöglichkeiten an Psychotherapeut:innen oder Mutter-Kind-Einrichtungen zur Verfügung stehen und die Versorgungsmöglichkeiten weiter ausgebaut werden sollten. Entsprechend der AWMF-Leitlinie zur unipolaren Depression würde sich auch im Bereich PPD ein Versorgungskonzept nach dem Collaborative-Care-Modell anbieten, welches durch eine engere Vernetzung der primären Versorger eine leitliniengerechte Behandlung ermöglichen kann [9].

Personenbezogenen Faktoren wie Schuld- und Schamgefühle, die zu einer mangelnden Inanspruchnahme der Unterstützungsangebote führen, könnte ebenfalls durch eine bessere Schulung der Versorger und somit einer verbesserten Psychoedukation begegnet werden. Zudem können internetbasierte Aufklärungsangebote, wie beispielsweise die Smartphone-Applikation „smart-moms“ [50], eine mögliche Unterstützung bei der Verbesserung der Versorgungssituation in Deutschland bieten.

Diese Übersichtsarbeit bietet einen berufsgruppenübergreifenden Überblick zum Thema Versorgung der PPD in Deutschland. Aufgrund der unterschiedlichen Methodiken der Teilstudien, mit teilweise verschiedenen Erhebungsinstrumenten und Items, lässt sich ein empirischer Vergleich der Versorger:innen nur eingeschränkt herstellen. Als weitere Limitationen der Studie müssen besonders die geringen Rücklaufquoten und die damit zu vermutenden Selektionseffekte in den Stichproben hervorgehoben werden: Die vorliegenden Studien zeigen gute (35 % Gynäkolog:innen) bis geringe Rücklaufquoten (8 % Hebammen, 7 % Hausärzt:innen). Die Vergleichbarkeit ist damit eingeschränkt und die vorliegenden Aussagen spiegeln unter Umständen nicht das allgemeine Meinungsbild aller Tätigen wider, da die Teilnahme an den Befragungen ein gewisses Interesse und Kooperationsbereitschaft auf dem Gebiet der psychischen Erkrankungen und an wissenschaftlichen Studien vermuten lässt. Es ist anzunehmen, dass Versorger:innen, die sich für die PPD zuständig fühlen und Symptome einer PPD im Patientengespräch oder mittels eines Screeningverfahrens erfassen, in unserer Stichprobe überrepräsentiert sind, da die Befragungen von Hebammen, Gynäkolog:innen und Hausärzt:innen eine freiwillige Teilnahme voraussetzten und somit vermehrt an dem Thema PPD interessierte und sensibilisierte Versorger:innen teilnahmen. Die Befragung der Vertreter:innen der BVKJ kann ebenfalls nicht als repräsentativ für alle niedergelassenen Pädiater:innen in Deutschland eingestuft werden, da gegebenenfalls berufspolitische Aspekte die Beantwortung der Fragen beeinflusst haben könnten. Auch ließ sich im Rahmen der qualitativen Befragung mit offen formulierten Fragen in einigen Fällen keine thematische Gruppierung der Antworten für einen Vergleich erfassen.

## Fazit

Zusammenfassend zeigt sich, dass auch wenn über alle Berufsgruppen hinweg ein persönliches und wirtschaftliches Interesse an einer geregelten Vergütung der Beratung besteht, der Schlüssel für eine bessere Versorgung von Frauen mit PPD in einer engeren Zusammenarbeit aller Zuweiser:innen und Versorger:innen liegt. So sollten die Identifikation und Zuweisung von Frauen mit PPD durch Aus- und Fortbildungen für Ärzt:innen und Hebammen und eine sichere Vermittlung im Rahmen eines interdisziplinären Versorgungsnetzwerks verbessert werden.

## Supplementary Information




## Footnotes

1 Als Wöchnerin wird eine Frau in den ersten 6–8 Wochen nach einer Geburt bezeichnet – eine Phase, in der sich ihre Geschlechtsorgane (außer Brüste) auf den Zustand vor der Schwangerschaft zurückbilden.

2 Die Perinatalzeit ist der Zeitraum zwischen der 28. Schwangerschaftswoche und dem 7. Tag nach der Geburt.
