# Supplementary material for: Postpartum depression—who cares? Approaches to care via midwifery, gynaecology, paediatrics and general practice
Source: Bundesgesundheitsblatt Gesundheitsforschung Gesundheitsschutz. 2022 May 12;65(6):658–67. [Article in German] doi: 10.1007/s00103-022-03545-8 (PMC9132831; doi:10.1007/s00103-022-03545-8)
Supplement: Supplementary file 1 [file 103_2022_3545_MOESM1_ESM.pdf]

Bundesgesundheitsblatt – Gesundheitsforschung – Gesundheitsschutz, Ausgabe 6/2022

Onlinematerial zum Beitrag:

Postpartale Depression – wer kümmert sich? Versorgungszugänge über Hebammen, Gynäkologie, Pädiatrie und Allgemeinmedizin

Silke Pawils[1*], Eileen Kochen[1*], Nora Weinbrenner[1], Viola Loew[1], Kornelia Döring[1], Daria Daehn[2], Claudia Martens[1], Philip Kaczmarek[1], Babette Renneberg[2]

[*] geteilte Erstautorenschaft

[1] Universitätsklinik Hamburg-Eppendorf, Zentrum für Medizinische Psychologie, Institut und Poliklinik für Medizinische Psychologie, Hamburg, Deutschland

[2] Freie Universität Berlin, Fachbereich Erziehungswissenschaften und Psychologie, Klinische Psychologie und Psychotherapie, Berlin, Deutschland

Korrespondenzadresse

PD Dr. Silke Pawils
Zentrum für Psychosoziale Medizin
Institut und Poliklinik für Medizinische Psychologie
Martinistraße 52
20246 Hamburg
Deutschland
s.pawils@uke.de

Tabelle mit Items und Antwortskalen aus drei Fragebögen zur quantitativen Befragung von Gynäkolog:innen, Hebammen und Hausärzt:innen zu deren persönlichen Zuständigkeitsgefühl für die Detektion und Intervention bei Postpartaler Depression (PDD)

**Gynäkolog:innen**	
Items (N = 28)	Antwortformat
**Soziodemografische Angaben zur Praxis und Person**	
Praxisart	Dichotome Nominalskala: „Einzelpraxis“ oder „Gemeinschaftspraxis“
Kassenzulassung	Dichotome Nominalskala: „ja (kassenärztlich)“ oder „nein (Privatpraxis)“
Bundesland	Freitextangabe
Stadt	Freitextangabe
Einwohnerzahl	Numerische Freitextangabe
Einzugsgebiet	Nominalskala: „eher städtisch", “eher ländlich” oder „beides”
Anzahl der betreuten Schwangeren pro Jahr (Scheine pro Quartal)	Numerische Freitextangabe
Geschlecht	Dichotome Nominalskala: „männlich” oder „weiblich“

Alter	Numerische Freitextangabe
Dauer der Berufstätigkeit	Numerische Freitextangabe
Zusatzqualifikationen	Dichotome Nominalskala mit Filterfunktion: „ja” oder „nein”; bei „ja”:
Ja „Wenn ja, welche:“	Freitextangabe
**Diagnostik und Umgang mit Patientinnen mit Verdacht auf PPD**	
Schätzung des Anteils der Patientinnen mit PPD innerhalb der Praxis	Numerische Freitextangabe in Prozent
Art der Ermittlung oder des Erfahrens von einer PPD	Nominalskala mit optionaler Mehrfachantwort:<br>• „im Gespräch, und zwar…” entweder „gezielt erfragt” oder „zufällig bzw. wenn es die Patientin selbst anspricht”<br>• „mittels Fragebogen, und zwar…“ entweder „routinemäßig” oder „bei Bedarf“<br>• durch Hinweise einer dritten Person (z.B. Hebamme, ArzthelferIn, Angehörige)
Alter der betroffenen Patientinnen mit PPD im Durchschnitt	Dreistufige Ordinalskala mit optionaler Mehrfachantwort: „sehr junge und junge Mütter (unter 20 Jahre)“, „Mütter im mittleren Alter (20 - 40 Jahre)“ oder „Frauen im höheren Alter (über 40 Jahre)“
Vorgehen nach Feststellung einer PPD bei einer Patientin	Vierstufige Ordinalskala mit optionaler Mehrfachantwort: „Beratung der Patientin”, „Vermittlung an eine spezialisierte Beratungsstelle”, „Überweisung an einen Therapeuten oder eine Klinik“ und „Sonstiges:“ mit Freitextangabe
Zuständigkeitsgefühl hinsichtlich der Erkennung von PPD bei Schwangeren/Patientinnen	Vierstufige Likert-Skala: „ja, auf jeden Fall”, „eher ja”, „eher nein” oder „nein, auf keinen Fall” mit Begründung in Form einer Freitextangabe
Schätzung des Anteils der Schwangeren mit psychosozialen, finanziellen oder psychischen Belastungen innerhalb der eigenen Praxis	Numerische Freitextangabe in Prozent
Art der Ermittlung oder des Erfahrens von Belastungssituationen	Nominalskala mit optionaler Mehrfachantwort:<br>• „im Gespräch, und zwar…” entweder „gezielt erfragt” oder „zufällig bzw. wenn es die Patientin selbst anspricht”<br>• „mittels Fragebogen, und zwar…“ entweder „routinemäßig” oder „bei Bedarf“<br>• durch Hinweise einer dritten Person (z.B. Hebamme, ArzthelferIn, Angehörige)
Themen, die außerhalb der medizinischen Betreuung im Kontakt mit der Schwangeren angesprochen werden	Vierstufige Likert-Skala: „oft“, „gelegentlich“, „selten” oder „nie” zur Beantwortung folgender Themenfelder:<br>„Einstellung zur Schwangerschaft“, „Partnerschaft“, „Psychische Gesundheit“, „Finanzielle Situation“ und „Belastungen des Partners“
Sich ergebende Belastungen von Schwangeren	Vierstufige Likert-Skala: „nie/selten”, „manchmal“, „oft” oder „sehr oft” mit optionaler Mehrfachantwort zur folgenden Auflistung möglicher Belastungen: „ungewollte Schwangerschaft”, „Konflikte in der Partnerschaft”, „Alltagsbewältigung (z.B. als Alleinerziehende)”, „geringe soziale Unterstützung”, „Schwierigkeiten mit der Sprache“, „Arbeitslosigkeit”, „Schulden”, „schlechte Wohnsituation”, „fehlende Krankenversicherung”, „psychische Probleme”, „Suchterkrankung“, „psychische Probleme oder Suchterkrankung des Partners“ und „sonstige Belastungen“ mit Freitextangabe
Alter der betroffenen Patientinnen mit psychosozialen, finanziellen oder psychischen Belastungen im Durchschnitt	Dreistufige Ordinalskala mit optionaler Mehrfachantwort: „sehr junge und junge Mütter (unter 20 Jahre)“, „Mütter im mittleren Alter (20 - 40 Jahre)“ oder „Frauen im höheren Alter (über 40 Jahre)“
Vorgehen bei der Feststellung von Hilfebedarf einer Patientin durch psychosoziale, finanzielle oder psychische Belastungen	Ordinalskala mit optionaler Mehrfachantwort: „Beratung der Patientin“, „Vermittlung an eine spezialisierte Beratungsstelle”, „Überweisung an einen Therapeuten oder eine Klinik“, „Vermittlung an ein Projekt der Frühen Hilfen“, „Vermittlung einer Hebamme“, „Vermittlung an eine Familienhebamme“ und „Sonstiges“ mit Freitextangabe
Zuständigkeitsgefühl hinsichtlich der Erkennung von psychosozialen Belastungen bei Schwangeren bzw. Patientinnen	Vierstufige Likert-Skala: „ja, auf jeden Fall”, „eher ja”, „eher nein” oder „nein, auf keinen Fall“ mit Begründung in Form einer Freitextangabe

**System- und patientenbezogene Barrieren im Management der Identifikation und Überleitung in Hilfen**	
Erschwerende Aspekte für die Beratung bzw. Weitervermittlung der Schwangeren / Mutter bei psychosozialen, finanziellen oder psychischen Belastungen	Liste für „Systembezogene Umstände“ mit optionaler Mehrfachantwort: „die zur Verfügung stehende Beratungszeit”, „die geringe Vergütung der Beratung”, „die fehlende Erstattung durch die Krankenkasse“, „das Fehlen effektiver Behandlungsmöglichkeiten / Betreuungsmöglichkeiten“ und „Qualität der Überweisungsmöglichkeiten und Kooperation (mit Beratungsstellen, Kliniken, Therapeuten, Krankenkassen)“ sowie die Liste zu „Patientenbezogenen Umständen“ mit optionaler Mehrfachantwort: „Ablehnen/Nichtinanspruchnahme der Überweisung“, „fehlende Akzeptanz der Diagnose durch Patientin oder Partner“ und „andere/ (z.B. somatische) Probleme stehen im Vordergrund“ oder „Sonstiges“ mit Freitextangabe
Notwendigkeit zur Verbesserung der Versorgungssituation für (werdende) Mütter mit psychosozialen, finanziellen oder psychischen Belastungen	Dichotome Nominalskala: „Ja“ oder „Nein“ mit einer Begründung als Freitextangabe
Ja – Verbesserung der Versorgungssituation	Liste mit fünf Auswahlmöglichkeiten: „Teilnahme an zertifizierten Fortbildungen”, „systematisches Nachfragen nach psychosozialen, finanziellen oder psychischen Problemen“, „enge Zusammenarbeit mit Kliniken, Beratungsstellen, Frühen Hilfen, Familienhebammen, Hebammen, Familienlotsen etc.“, „Erstattung der Beratung durch Krankenkassen“ oder „Sonstiges“ mit Freitextangabe sowie einer Freitextangabe als Bemerkung
**Hebammen**	
Items (N = 15)	Antwortformat
**Soziodemografische Daten der Hebamme**	
Arbeitsverhältnis	Dichotome Nominalskala: „Freiberuflich“ oder „Angestellt bei“ mit Freitextangabe
Bundesland	Freitextangabe
Einzugsgebiet	Nominalskala: „Eher städtisch", “Eher ländlich” oder „Beides”
Anzahl der betreuten Schwangeren pro Jahr	Numerische Freitextangabe
Geschlecht	Dichotome Nominalskala: „männlich” oder „weiblich“
Dauer der Berufstätigkeit	Numerische Freitextangabe
Zusatzqualifikationen	Dichotome Nominalskala mit Filterfunktion: „ja” oder „nein”; bei „ja”: Freitextangabe
**Vorwissen und der bisherige Umgang mit PPD-betroffenen Müttern**	
Schätzung des Anteils von Patientinnen mit PPD in der Hebammenpraxis	Numerische Freitextangabe in Prozent
Art der Ermittlung oder des Erfahrens von PPD bei einer Schwangeren	Nominalskala mit optionaler Mehrfachantwort:<br>• „Im Gespräch, und zwar…” entweder „Gezielt erfragt” oder „Zufällig bzw. wenn es die Patientin selbst anspricht”<br>• „Mittels Fragebogen, und zwar…“ entweder „Routinemäßig” oder „Bei Bedarf“<br>• Durch Hinweise einer dritten Person (Medizinisches Personal, Angehörige)
Alter der betroffenen Patientinnen mit PPD im Durchschnitt	Dreistufige Ordinalskala mit optionaler Mehrfachantwort: „sehr junge und junge Mütter (<20 J.)“, „Mütter im mittleren Alter (20-40 J.)“ oder „Frauen im höheren Alter (> 40 J.)“
**Intervention bei Verdacht auf PPD**	
Vorgehen bei Vermutung einer PPD bei einer Patientin	Vierstufige Ordinalskala mit optionaler Mehrfachantwort: „Beratung der Patientin”, „Vermittlung an eine spezialisierte Beratungsstelle”, „Überweisung an einen Therapeuten oder eine Klinik”, „Vermittlung an ein Projekt der Frühen Hilfen“, „Vermittlung an einen Gynäkologen“ und „Sonstiges:“ mit Freitextangabe
**Zuständigkeitsgefühl bezüglich Patientinnen mit PPD in der Hebammenhilfe**	
Zuständigkeitsgefühl hinsichtlich der Erkennung von PPD bei den Schwangeren / Patientinnen	Vierstufige Likert-Skala: „ja, auf jeden Fall”, „eher ja”, „eher nein” oder „nein, auf keinen Fall“ mit Begründung in Form einer Freitextangabe

**Derzeitige Versorgungssituation der PPD in der Hebammenhilfe**		
Erschwerende Aspekte für die Beratung bzw. Weitervermittlung der Mutter bei einer PPD		Liste für „Systembezogene Umstände“ mit optionaler Mehrfachantwort: „Die zur Verfügung stehende Beratungszeit”, „Die Vergütung der Beratung”, „Die fehlende Erstattung durch die Krankenkasse“, „Das Fehlen effektiver Behandlungsmöglichkeiten/Betreuungsmöglichkeiten“, „Qualität der Überweisungsmöglichkeiten und Kooperation (mit Beratungsstellen, Kliniken, Therapeuten, Krankenkassen)“, „Ablehnen/Nichtinanspruchnahme der Überweisung“, „fehlende Akzeptanz der Diagnose durch Patientin oder Partner“ und „andere (z.B. somatische) Probleme stehen im Vordergrund“ oder „Sonstiges“ mit Freitextangabe
**Mögliche Anregungen für die zukünftige Versorgung der PPD**		
Notwendigkeit zur Verbesserung der Versorgungssituation für Mütter mit PPD		Dichotome Nominalskala: „Ja“ oder „Nein“ mit einer Begründung als Freitextangabe
Ja	Verbesserung der Versor-gungssituation	Liste mit fünf Auswahlmöglichkeiten: „Teilnahme an zertifizierten Fortbildungen”, „Systematisches Nachfragen nach PPD“, „Enge Zusammenarbeit mit Kliniken, Beratungsstellen, Frühen Hilfen, Gynäkologen, Familienlotsen, Hausärzten etc.“, „Erstattung der Beratung durch Krankenkassen“, „Einsatz eines Fragebogens zur systematischen Diagnostik der Postpartalen Depression” oder „Sonstiges“ mit Freitextangabe sowie einer Freitextangabe als Bemerkung
**Hausärzt:innen**		
Items (N = 21)		Antwortformat
**Vorkommen von betroffenen Frauen in der hausärztlichen Praxis**		
Behandlung von einer oder mehreren Patientinnen mit PPD im letzten Jahr innerhalb der Praxis		Dichotome Nominalskala: „Ja“ oder „Nein“
Ja	Einschätzung der Anzahl behandelter Patientinnen mit PPD im letzten Jahr innerhalb der Praxis	Vierstufige Ordinalskala mit Kategorien: „1 – 2“, „3 – 4, „5 – 10“ oder „> 10”
**Selbsteinschätzung bzgl. des Wissensstandes über das Krankheitsbild der PPD und Erfragung des Verantwortungsgefühls**		
Empfundener Wissensstand bezüglich des Krankheitsbildes der PPD		Dreistufige Likert-Skala: „Ja, ich habe ausreichend Wissen“ mit Freitextangabe zu „Woher: Studium, Fortbildung?“, „Nein, ich hätte gerne mehr Wissen darüber“ mit Freitextangabe zu Beispielen oder „Ich halte umfangreiches Wissen über die PPD in meinem Tätigkeitsbereich für nicht notwendig.“
Erkennung von PPD als Aufga-benbereich von Hausärzt:innen		Vierstufige Likert-Skala: „Ja, auf jeden Fall.“, „Eher ja.“, „Eher nein.“ und „Nein“ mit Freitextangaben zur Begründung
**Informationen und Hintergrundwissen zur PPD: Wissen über Prävalenz und Leitlinien in Deutschland**		
Schätzung der Prävalenz von Frauen im Wochenbett mit PPD in Deutschland		Numerische Freitextangabe in Prozent
Bekanntheit der Empfehlungen der AWMF S3-Leitlinie „Unipolare Depression“ für die Behandlung der PPD		Dichotome Nominalskala: „Ja, sie sind mir bekannt.“ oder „Nein, sie sind mir nicht bekannt.“
**Umgang mit betroffenen Frauen: Screening, Behandlung und Erfahrungen bei der Weitervermittlung**		
Ermittlung von Anzeichen einer PPD bei Patientinnen		Vierstufige Skala mit optionaler Mehrfachantwort: „Mithilfe eines Fragebogens”, „Im Gespräch/Interview", „Durch andere Methoden:“ oder „Ich erfasse keine Anzeichen einer PPD.“ mit Freitextangaben
Merkmale der Frauen, bei welchen Anzeichen einer PPD ermittelt werden		Sechsstufige Ordinalskala mit optionaler Mehrfachantwort: „Bei jeder Frau in der Schwangerschaft“, „Bei jeder Frau nach der Geburt.“, „Bei Frauen, die mir nach der Geburt depressiv erscheinen.“, „Bei Frauen, die das Thema selbstständig ansprechen.“, „Ich erfasse keine Anzeichen einer PPD“ oder „Sonstiges:“ mit Freitextangabe
Vorgehen bei Verdacht auf eine PPD bei einer Patientin		Fünfstufige Ordinalskala mit optionaler Mehrfachantwort: „Ich führe selbst eine Beratung durch.”, „Ich vermittle die Frau an weiterführende Hilfs-/Beratungsangebote”, „Ich vermittle die Frau an eine Therapeut*in/ eine Klinik“, „Ich biete psychosoziale Unterstützung z.B. durch eine Familienhebamme oder eine Haushaltshilfe an.“ oder „Ich ergreife keine weiteren Maßnahmen, weil“ mit Freitextangaben

Gelingen von Vermittlung der Patientinnen an Therapie/Klinik und Hilfs- bzw. Beratungsangebote	Dichotome Nominalskala: „Ja, mir gelingt die Vermittlung meiner Patientinnen.“ oder „Ich versuche die Frau weiter zu vermitteln, dies gelingt mir jedoch nicht immer.“ mit Freitextangabe
**Durch Hausärzt:innen wahrgenommene Barrieren in der Versorgung von Frauen mit PPD**	
Barrieren für Frau mit PPD nach Beratung und Weitervermittlung	Liste für „Systembezogene Umstände“ mit optionaler Mehrfachantwort: „die begrenzte Beratungszeit“, „die geringe Vergütung der Beratung“, „die fehlende Erstattung durch die Krankenkasse“, „das Fehlen effektiver Behandlungsmöglichkeiten / schwierige Weitervermittlung“, “die langen Wartezeiten für Psychotherapieplätze“ und „die unzureichende interdisziplinäre Zusammenarbeit z.B. Hebammen oder Gynäkolog*innen“ sowie die Liste zu „Patientenbezogenen Umständen“ mit optionaler Mehrfachantwort: „Ablehnen / Nichtinanspruchnahme der Überweisung“, „Fehlende Akzeptanz der Diagnose durch Patientin / Partner*in“, „Angst vor Stigmatisierung“ und „Andere/ (z.B. somatische) Probleme stehen im Vordergrund“
**Einschätzung bzgl. geeigneter Formen der Informationsvermittlung für die Aufklärung von Patientinnen über das Krankheitsbild der PPD**	
Geeignete Formen der Informa-tionsvermittlung für Patientinnen	Vierstufige Ordinalskala mit optionaler Mehrfachantwort: „Bücher / Literaturempfehlung“, „Flyer“, „Selbsthilfegruppen“ und „Verweise auf digitale Informationsangebote“
Empfehlung einer App zur Informationsvermittlung hinsichtlich PPD und Inanspruchnahme eines Screenings	Dreistufige Likert-Skala: „Ja, das kann ich mir gut vorstellen“, „Ich bin mir nicht sicher.“ oder „Nein, das kann ich mir nicht vorstellen.“ mit Freitextangaben
**Soziodemografische Angaben zu Praxis und Person**	
Praxisart	Nominalskala: „Einzelpraxis“, „Praxisgemeinschaft“, „Medizinisches Versorgungszentrum“ oder „Berufsausübungsgemeinschaft / Gemein-schaftspraxis”
Kassenzulassung	Dichotome Nominalskala: „kassenärztlich“ oder „rein privatärztlich“
Bundesland	Nominalskala: „Hamburg“, „Schleswig-Holstein“, „Mecklenburg-Vorpommern“ oder „Niedersachsen“
Einzugsgebiet	Nominalskala: „eher städtisch", “eher ländlich” oder „beides”
Geschlecht	Nominalskala: „männlich”, „weiblich“ oder „divers“
Alter	Fünfstufige Ordinalskala mit Kategorien: „unter 30“, „30 – 40“, „40 – 50“, „50 – 60“ oder “> 60 Jahre“
Dauer der Berufstätigkeit	Numerische Freitextangabe
Zusatzqualifikationen	Dichotome Nominalskala mit Filterfunktion: „Ja” oder „Nein”; bei „Ja”: Freitextangabe
